# Comprehensive cost-effectiveness of diabetes management for the underserved in the United States: A systematic review

**DOI:** 10.1371/journal.pone.0260139

**Published:** 2021-11-18

**Authors:** Rita Bosetti, Laila Tabatabai, Georges Naufal, Terri Menser, Bita Kash

**Affiliations:** 1 Center for Outcomes Research, Houston Methodist, Houston, Texas, United States of America; 2 Division of Endocrinology, Diabetes & Metabolism, Houston Methodist, Houston, Texas, United States of America; 3 Public Policy Research Institute, Texas A&M University, College Station, Texas, United States of America; 4 Department of Population Health Sciences, Weill Cornell University, New York, New York, United States of America; 5 School of Public Health, Texas A&M University, College Station, Texas, United States of America; Icahn School of Medicine at Mount Sinai, UNITED STATES

## Abstract

**Background:**

Diabetes mellitus affects almost 10% of U.S. adults, leading to human and financial burden. Underserved populations experience a higher risk of diabetes and related complications resulting from a combination of limited disposable income, inadequate diet, and lack of insurance coverage. Without the requisite resources, underserved populations lack the ability to access healthcare and afford prescription drugs to manage their condition. The aim of this systematic review is to synthesize the findings from cost-effectiveness studies of diabetes management in underserved populations.

**Methods:**

Original, English, peer-reviewed cost-effectiveness studies of diabetes management in U.S. underserved populations were obtained from 8 databases, and PRISMA 2009 reporting guidelines were followed. Evidence was categorized as strong or weak based on a combination of GRADE and American Diabetes Association guidelines. Internal validity was assessed by the Cochrane methodology. Studies were classified by incremental cost-effectiveness ratio as very cost-effective (ICER≤US$25,000), cost-effective (US$25,000<ICER≤US$50,000), marginally cost-effective (US$50,000<ICER≤US$100,000) or cost-ineffective (ICER>US$100,000). Reporting and quality of economic evaluations was assessed using the CHEERS guidelines and Recommendations of Second Panel for Cost-Effectiveness in Health and Medicine, respectively.

**Findings:**

Fourteen studies were included. All interventions were found to be cost-effective or very cost-effective. None of the studies reported all 24 points of the CHEERS guidelines. Given the considered cost categories vary significantly between studies, assessing cost-effectiveness across studies has many limitations. Program costs were consistently analyzed, and a third of the included studies (n = 5) only examined these costs, without considering other costs of diabetes care.

**Interpretation:**

Cost-effectiveness studies are not based on a standardized methodology and present incomplete or limited analyses. More accurate assessment of all direct and indirect costs could widen the gap between intervention and usual care. This demonstrates the urgent need for a more standardized and comprehensive cost-effectiveness framework for future studies.

## Introduction

Diabetes mellitus (DM) affected 30.3 million U.S. adults in 2015, and an estimated 1.5 million new cases of DM annually makes diabetes the most common chronic disease in the U.S. [1]. This is reflected in the U.S. burden for DM, estimated at US$327 billion in 2017 [2]. The highest prevalence of DM and its complications are found among the uninsured, patients with lower socio-economic status and racial minorities [3]. The daily management of type 2 diabetes mellitus (T2DM) is burdensome and costly, since–if uncontrolled–T2DM leads to severe, long-term microvascular and macrovascular complications, such as cardiovascular disease, stroke, eye disorders, foot ulcers, and chronic kidney disease [4, 5]. T2DM is the most common cause of end-stage renal disease (ESRD) in the U.S. [6], which accounts for a significant percent of total Medicare budget. ESRD accounts for US$33.9 billion– 7.1% of Medicaid paid claims in 2015 [7]. In the challenge of effectively treating T2DM, it is important to produce near-normal glucose levels (HbA1C) [8].

Support given to diabetes patients is a first step in the public health approach to successfully managing the disease and preventing complications. Many initiatives are considering improved care for underserved populations, although the real cost of such interventions has not been assessed. By underserved populations, we refer to populations that face barriers and challenges in accessing and using resources due to residence in impoverished urban sectors, poverty or low socio-economic status, the uninsured, persons from disadvantaged backgrounds, or individuals with low income [9]. The use of limited resources requires understanding of the cost-effectiveness of interventions–the ratio of the difference in costs to the difference in effectiveness between intervention and usual care, or incremental cost-effectiveness ratio (ICER). Several reviews of cost-effectiveness studies of diabetes interventions can be found [10–14], but none focus on underserved populations experiencing severe disparities in the incidence of DM. Underserved populations often have limited access to specialty care. Therefore, diabetes management for these populations is often offered in the form of self-management. Diabetes self-management refers to the activities and behaviors an individual undertakes to control and treat their condition. It includes the regular monitoring of their health, which typically occurs at home. This study focuses on the cost-effectiveness of diabetes management in underserved populations. It provides summary information to guide diabetes programs in underserved populations, and assesses the methodological rigor of cost-effectiveness studies, in order to open a dialogue encouraging more comprehensive economic evaluations. A review focusing on underserved populations in the United States is necessary, since this population has limited access to specialty care as well as prescription drugs due to lack of insurance or underinsurance. These individuals often receive totally different care than insured individuals with full access to specialty care. Diabetes management for underserved individuals is mostly based on self-management. Consequently, underserved populations not only have higher risks from type 2 diabetes but also increased risk of uncontrolled type 2 diabetes and related long-term complications.

## Methods

### Data sources and searches

We followed the Preferred Reporting Items for Systematic Reviews and Meta-Analyses (PRISMA) reporting guidelines [15], and a scientifically accepted search strategy [16]. We searched MEDLINE (Ovid), PubMed, EBSCOhost, NHS and Economic Evaluation Database, Health Technology Assessment, ISI Web of Science, Scopus, and Google Scholar from inception to December 21, 2020. Original articles measuring the cost-effectiveness of diabetes management in underserved adult populations (≥ 18 years of age) in the U.S. were searched. A broad strategy was used to search the databases, using both controlled terms (e.g., MeSH headings) and free keywords. One search strategy was developed for all databases (S1 File). Additional search terms were included in consultation with an information specialist. The following keywords were used: (community clinic OR community OR Federally Qualified Health Center OR integrated care OR underserved OR underinsured OR uninsured OR underinsurance OR Medicaid OR low income OR poor OR specialty access OR specialty care) AND (endocrine OR endocrinology OR diabetes OR type 2) AND (economic evaluation OR cost-benefit OR cost-effectiveness OR cost-utility OR economic analysis OR health economic). Searches were based on matches in all three keyword fields. References were screened to identify additional studies.

### Study selection

Two independent reviewers screened titles and abstracts, followed by retrieval and screening of full texts. Eligibility was based on the following criteria: original cost-effectiveness studies focusing on DM that include 1) lack of disposable income, insurance status or socio-economic status and a measure of cost assessment; 2) all adult type 2 diabetes management interventions–both interventions for diabetes prevention and diabetes care; 3) any publication date–from inception to the search date of December 21, 2020; 4) published literature concerning the U.S. only; and 5) any type of model used–meaning all cost-effectiveness studies meeting the inclusion criteria are included, regardless of the model to predict costs and effectiveness of the interventions based on the best available evidence. Short-term and long-term studies and studies from the payer or societal perspective were included. Finally, analyses with and without control groups are taken into account. Conference abstracts were excluded for providing insufficient detail. Studies published in languages other than English, reviews and unpublished studies (grey literature) were excluded. Therefore, additional web-based platforms–such as google searches for grey literature and the World Health Organization Global Health Library–were not searched. Care could be delivered by any provider type and could be individual- or group-based. Studies could be of any duration and intensity. Disagreements were resolved by consensus.

### Data extraction and reporting the results

Data extraction was based on the Cochrane Consumers and Communication Review Group’s data extraction template: (1) publication year; (2) journal; (3) population; (4) sample size; (5) intervention; (6) study design; (7) clinical and economic outcomes. Full text review of relevant articles and reference crawling was carried out. Missing information was sought from the authors. Data extraction was unblinded since blinding does not decrease bias in systematic reviews [17, 18]. A second reviewer checked the extracted data. Disagreements were resolved by consensus.

Data were charted as follows: 1) results for each study were summarized, both key features and compliance with the Recommendations of the Second Panel on Cost-Effectiveness in Health and Medicine [19], and 2) each study was synthesized according to the classification and quality criteria described in the next section. The studies were grouped into two categories: diabetes prevention among high-risk individuals and diabetes management. Diabetes management is subdivided into: diabetes management through community health workers (CHWs), telephone-based diabetes management, diabetes self-management training, nurse case and peer education diabetes management, and quality improvement collaborative diabetes. It is important to note that studies were not always mutually exclusive and were subdivided based on the type of intervention. Also, due to the descriptive character of this manuscript, studies were placed only in one category. For example, although some CHWs had telephonic contact with the patients, the studies placed in the CHW category were focused on the intervention of a CHW–a person with the same background and disease history as the patients–to help patients improve their HbA1C. It was irrelevant if the CHW went to the patient in person or talked to the patient remotely (telephone). For the telephonic interventions, interventions giving patients additional training over the telephone, in comparison with no additional training, were included. Multiple-study interventions, focusing on different populations or study designs, are included since they generate different results.

The studies’ results were synthesized as follows: 1) for multiple-study interventions, the median ICER was reported; and 2) for studies reporting short- and long-term results, only long-term results were reported, since outcomes are mostly long-term in nature. To make outcomes across studies comparable, costs are expressed as 2019 dollars, using the Consumer Price Index [20]. For studies not mentioning the year of cost calculation, we assumed the year before publication.

### Classification and quality of cost-effectiveness studies

Interventions are classified based on cost-effectiveness result [21]. An intervention may be cost-saving (better outcomes and costs), or cost-effective (better outcomes at higher costs) [21]. If cost-effective, interventions can be classified as: 1) very cost-effective (ICER ≤ US$25,000); 2) cost-effective (US$25,000 < ICER ≤ US$50,000); or 3) marginally cost-effective (US$50,000 < ICER ≤ US$100,000). If the ICER > US$100,000, the intervention is considered cost-ineffective. Study quality is based on whether the evidence for the intervention’s cost-effectiveness was strong (i.e., high confidence in the estimate) or weak (i.e., further research to confirm the estimate) [22]. Quality of the evidence is based on four elements: study design, study quality, consistency, and directness [23, 24]. Table 1 gives an overview of the classification of studies based on the quality of their evidence according to a combination of GRADE [23] and American Diabetes Association (ADA) standards [24]. GRADE/ADA criteria were chosen to check the quality of the underlying evidence used in cost-effectiveness studies. Cost-effectiveness studies can only be as good as the underlying data. If the data is not of excellent quality, the cost-effectiveness studies cannot be of excellent quality either.

**Table 1:** Classification and quality of studies.

Level of evidence	
Strong	Results based on:
	• Well-conducted randomized controlled trial **OR** two or more observational studies [23]
	**AND EITHER:**
	• Excellent study quality [23, 24]
	• All confounders have been considered
	• Good internal validity
	**OR**
	• American Diabetes Association (ADA) level A or level B evidence [24]
Weak	Results are based on [23, 24]:
	• Less than excellent study quality evidence
	• Inconsistencies
	• Imprecise or sparse data
	• High probability of bias
	• ADA’s level C evidence

Two independent reviewers appraised the reporting and quality of the economic component of the studies using the Consolidated Health Economic Evaluation Reporting Standards (CHEERS) [25] checklist and Recommendations of the Second Panel on Cost-Effectiveness in Health and Medicine [19]. Cost-effectiveness studies pose a particular challenge for reporting because substantial information must be used to allow scrutiny of study findings. Therefore, the International Society for Pharmacoeconomics and Outcomes Research (ISPOR) proposed the Consolidated Health Economic Evaluation Reporting Standards (CHEERS) to improve reporting in a user-friendly manner. The CHEERS checklist consists of 24 items, subdivided in six main categories: title and abstract, introduction, methods, results, discussion, and other (S1 Table). Excellent cost-effectiveness studies should thus be based on excellent evidence but should also be reported in a clear way. Therefore, the quality of existing cost-effectiveness studies is graded using both GRADE/ADA criteria as well as CHEERS guidelines. Disagreements between coders were resolved by discussion.

### Validity

Internal validity was checked using the Cochrane methodology [26] (Table 2). Two independent reviewers determined randomization, allocation of concealment (selection bias), blinding of patients and personnel (performance bias), blinding of outcome assessment, incomplete outcome data, and selective reporting. Since these criteria are too strict in T2DM [27], we modified them. Since most studies did not comment on method of allocation and blinding patients is often impossible, allocation concealment and patient blinding were discarded as validity criteria [27, 28]. Also, an attrition rate of 20% or more was considered a potential source of bias [27].

**Table 2:** Tool to assess internal validity based on the Cochrane methodology [26].

Type of bias	How it is defined in our analysis
Selection bias	At baseline, there are systematic differences between control and intervention group
	Prevention:
	• Randomization
	• No significant differences between control and intervention groups on all variables OR in case of existing differences there should be an adequate statistical consideration for confounding
Performance bias	Systematic differences between control and intervention group exist due to the care provided
	Prevention:
	• No existing contamination or co-intervention
	• No contact with providers for the individuals in the intervention group compared to control group
Attrition bias	Systematic differences exist due to drop-outs
	Prevention:
	• Attrition<20%
Detection bias	Systematic differences exist in outcomes assessments between intervention and control group
	Prevention:
	• blinding is required for every outcome subject to assessor interpretation

Finally, we assessed the studies’ external validity, considered adequate if the study population represented the target population. Another requirement was random subject selection or referral-based selection without significant differences between groups at baseline.

## Results

**Fig 1:**
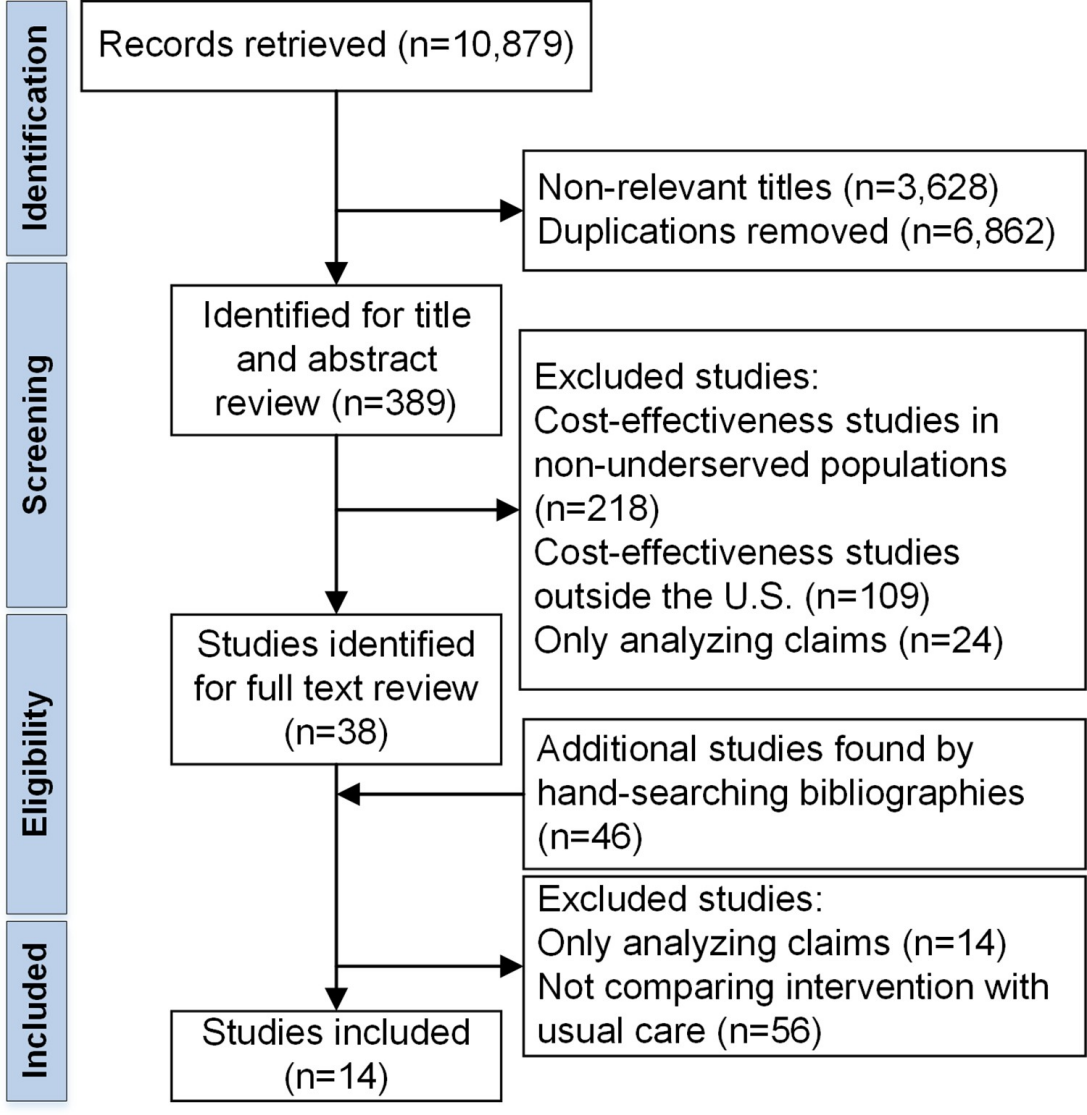
PRISMA flow chart.

The search resulted in 10,879 screened titles. After exclusion of non-relevant titles (n = 3,628) and duplicates (n = 6,862), 389 potentially relevant articles were independently reviewed. Among these, 351 studies were discarded since they did not meet the inclusion criteria. One-hundred nine studies examined cost-effectiveness in non-U.S. settings, 218 studies focused on non-underserved populations, and 24 studies analyzed claims. Forty-six additional studies were identified for full text review by hand-searching references. After full text review, an additional 70 studies were non-eligible because they analyzed only claims (14 studies) or the intervention was not compared to usual care (56 studies). Thirteen discrete studies, published in 14 articles, met the inclusion criteria. Fig 1 depicts the study flow diagram.

S2 Table shows an overview of the analyses included in the review according to intervention type [29–42]. The following information was used to describe the analyses: population, sample, intervention, control, study design, clinical outcomes, costs included, economic outcomes, perspective, analytical time horizon, discount rate, and cost-effectiveness results. Table 3 shows the results of the quality assessments and adherence to the CHEERS guidelines.

**Table 3:** Summary of the cost-effectiveness results according to intervention.

Intervention	Comparator	Population	Number of studies	Evidence	Median of the cost-effectiveness results	Range of the cost-effectiveness results	Cost-effectiveness in 2019 dollars	Comments
Diabetes Prevention Program Lifestyle Intervention adapted for community settings—Intensive group-based lifestyle intervention	No control	Medicaid beneficiaries at high risk for type 2 diabetes	1	Weak	US$14,011/QALY	US$9,998/QALY-US$312,063/QALY	US$15,116.51/QALY	• Differences in baseline between intervention and control group • No information about attrition • No information about patient blinding
Group Lifestyle Balance Program–Group-based sessions to achieve and maintain weight loss and to progressively raise activity levels to 150 minutes per week of moderately intense physical activity	Usual care	Urban, medically underserved population	1	Weak	US$3,420/QALY	US$0/QALY-US$18,600/QALY	US$5,078.36/QALY	• Improvement in control group with no intervention • No information about baseline characteristics • No information about attrition • No information about patient blinding
Home-based community health worker visits, classroom health education classes, nutrition classes, exercise classes, and counseling sessions	Hypothetical usual care	Low-income Hispanic adults	1	Weak	US$33,319/QALY	US$2,156/QALY-US$51,462/QALY	US$39,063.13/QALY	• No drop-outs • No information about patient blinding • Study population not representative for target population
Community health workers visiting patients at home for diabetes management	Waitlisted and standard care	Samoan population, low-income	1	Strong	US$13,191/QALY	US$13,191.24/QALY-US$74,750.36/QALY	US$14,690.77/QALY	• Randomization • Attrition: 9% • No significant differences between groups at baseline, except smoking • No information on patient blinding
Received a one-to-one culturally tailored diabetes education and management program along with usual care	Usual care	Low-income, uninsured, ethnic minority populations	1	Strong	US$355/QALY	Cost-saving-US$55,061/QALY	US$395.35/QALY	• Randomization • No information about attrition • Sample not representative for target population • No information about patient blinding
Received monthly visits from community health workers	Usual care	Poor or medically indigent immigrant population	1	Weak	US$13,810/QALY	N/A	US$16,401.38/QALY	• No randomization but participants are subject-matched • No drop-outs • No significant differences between groups at baseline • No information about patient blinding
Interactive phone technology to provide surveillance, patient education, and one-on-one counseling	Usual care	Low-income patients in safety-net clinics	1	Strong	US$32,333/QALY (US$65,167/QALY when start-up costs are considered)	US$29,402/QALY-US$72,407/QALY	US$39,882.05/QALY (US$80,382.06/QALY with start-up costs)	• Randomization • No differences between groups at baseline • Attrition: 10% • Sample population not representative for target population
Health educator for up to 10 self-management support phone calls to discuss self-management as found in the print materials mailed to them	Only print materials	Low-income, urban populations	1	Strong	US$2,617.35/additional person achieving HbA1C goal	US$1,483.52/additional person achieving HbA1C goal-US$10,826.14/additional person achieving HbA1C goal	US$2,975.80/additional person achieving HbA1C goal	• Randomization • No information about attrition • No differences between groups at baseline • No information about patient blinding • Sample not representative for target population
4 or 8 telephone calls over 12 months, depending on HbA1C level, from trained, supervised health educators to deliver behavioral counseling and self-management support, in addition to the print material	Only print materials	Low-income population	1	Weak	US$464.41/percentage point HbA1C	US$372.16/percentage point HbA1C-US$601.07/percentage point HbA1C	US$509.87/percentage point HbA1C	• Randomization • Attrition: 26% • No differences between groups at baseline • No information about patient blinding
Nurse-led team with registered nurse, certified diabetes educator, medical assistant and dietician. The goal is to meet the ADA standards of care and achieve improvements in HbA1C, blood pressure and lipid parameters. In addition, the program offers group self-management training (8 weeks) led by trained peer educators	Historical cohort of patients enrolled prior to the implementation of Project Dulce	Low-income, underinsured Latin	2	Weak	US$34,762.5/QALY	US$8,768/QALY-US$135,613/QALY	US$44,091.13/QALY	• Attrition: 12% • Some differences between groups at baseline • No information about patient blinding • Sample not representative for the target population
Patients received self-management support and group visits	No control	Patients of a community health center	1	Weak	US$33,386/QALY	US$23,653/QALY-US$416,850/QALY	US$45,192.17/QALY	• No control group • Incomplete information about patients and their care • Sample population may not be representative for the target population
Diabetes self-management training program with group classes and individual dietician consults	No control	Patients below the U.S. Federal poverty level	1	Weak	US$185/ decrease of 1.5 points in HbA1C	N/A	US$257.09/decrease of 1.5 points in HbA1C	• No randomization • No control group • Attrition: 32%
Various approaches are used in the different communities to reach and engage their respective patient populations in self-management	Usual care	Variety of ethnic populations in disadvantaged areas with notable health disparities	1	Strong	US$39,563/QALY	US$11,850/QALY-US$229,364/QALY	US$46,986.80/QALY	• Attrition: 13% • Sample is representative for the target population

ADA, American Diabetes Association; QALY, quality-adjusted life years.

### Characteristics of included studies

The evaluated interventions are to prevent T2DM in high-risk populations (2 studies) [36, 38], and/or to manage T2DM (12 studies) [29–35, 37, 39–42]. In the latter, we distinguish: diabetes management through CHWs (4 studies) [31, 32, 35, 41], diabetes self-management training (2 studies) [34, 37], telephonic diabetes self-management (3 studies) [39, 40, 42], nurse case and peer education diabetes management (2 studies) [29, 30], and Quality Improvement Collaborative diabetes (1 study) [33].

Seven of 14 studies evaluated the cost-effectiveness of diabetes interventions from a long-term analytical time horizon [30–36], with lifetime as the longest time horizon in which patients were followed (3 studies) [31, 33, 34]. These studies used simulation modeling to analyze long-term outcomes. Ten studies analyzed cost-effectiveness from the payer perspective [29–31, 34–37, 39, 40, 42]; only 4 studies applied the societal perspective [32, 33, 38, 41]. Furthermore, 1 of 14 studies included multiple comparisons in the analysis–comparing the cost-effectiveness of diabetes management for the uninsured, governmental, and commercial insurance [30]. Three studies calculated the ICER for subpopulations (2 studies) [32, 35] or sub-treatments (1 study) [33]. Four analyses were small (mean 53 subjects) [31–33, 37]. Most studies were not transparent about patient recruitment (9 studies) [30, 33–38, 40, 41].

### Quality of the included studies

Classification of the interventions based on the quality of their evidence and adherence to CHEERS reporting guidelines is presented in Table 3. For multiple-study interventions, the number of studies, the comparator, and study population are mentioned. Median and the range of the ICERs are presented.

Table 4 presents adherence to the Recommendations of the Second Panel on Cost-effectiveness in Health and Medicine [19], which aims at improving the quality of cost-effectiveness studies, recommending use of the societal perspective and quality-adjusted life years (QALYs). Accounting for productivity losses is also recommended [19]. No studies followed these recommendations. Four studies applied the societal perspective [32, 33, 38, 41]. One study considered indirect productivity losses [41]. Six studies did not consider the interventions’ direct medical costs [31, 37–42]. Six studies did not account for patients’ quality of life [29, 31, 37, 39–41]. Four studies failed to follow the recommendations [31, 37, 39, 40]. Finally, not all analyses were transparent about how cost-effectiveness was performed, since four studies [33–35, 38] did not give information about which cost categories were included.

**Table 4:** Cost calculation for the studies included in the review.

Author (year)	Perspective	Economic outcomes	Costs included	Adherence Recommendations Second Panel for Cost-effectiveness in Health and Medicine
Gilmer et al. (2018) [36]	Payer	Program costs, healthcare costs	**Program costs** (program coaching costs [staffing costs and costs for delivering the program curriculum], clinic costs [cost of program materials—measuring cups, exercise band, home scales, paper and other materials, educational materials, resources and services to support participation such as transportation and childcare], financial incentive costs [participation and goal-based incentive provided to program participants]); **healthcare costs** (costs for profession, outpatient, and pharmacy services, costs of complications)	**NO**
				• Direct medical costs
				• **No productivity losses**
				• QALYs
				• **No societal perspective**
Roberts et al. (2010) [38]	Societal	Program costs and costs of diabetes care	**Program costs** (costs of screening, personnel costs)	**NO**
				• **No direct medical costs**
				• **No productivity losses**
				• QALYs
				• Societal perspective
Brown et al. (2012) [32]	Societal	All measurable opportunity costs	**Program costs** (staff and volunteer time, participant time, materials, transport costs, fixed cost per class, community health worker training cost); **direct medical costs; and lifestyle change costs**	**NO**
				• Direct medical costs
				• **No productivity losses**
				• QALYs
				• Societal perspective
Huang et al. (2019) [41]	Societal	Program costs and costs of diabetes care	**Program costs** (start-up capital costs, staff salaries, donated space, other overhead costs); **costs for diabetes care** (clinic and hospital ambulatory costs, hospital emergency department costs, hospital inpatient costs, hospital procedure costs, patient indirect costs)	**NO**
				• Direct medical costs
				• Indirect patient costs (lost time)
				• Societal perspective
				• **No QALYs**
Prezio et al. (2014) [35]	Payer	Program costs and medical costs	**Program costs** (staff and participant time, supplies for CoDE program; **medical costs** (based on Archimedes model)	**NO**
				• Direct medical costs
				** • No productivity losses**
				• QALYs
				** • No societal perspective**
Ryabov (2014) [31]	Payer	Program costs	**Program costs** (cost of implementing the intervention (wages and office operations), records of materials and equipment used)	**NO**
				• No direct medical costs
				**• No productivity losses**
				**• No societal perspective**
				**• No QALYs**
Handley et al. (2008) [42]	Payer	Program costs	**Program costs** (nurse care manager training, development of ATSM messages, translation and recording of messages in 3 languages, programming setup costs, patient recruitment and follow-up time, fixed monthly ATSM maintenance, costs associated with outgoing weekly ATSM calls, and direct nurse telephone care management with patients, and overhead costs)	**NO**
				**• No direct medical costs**
				**• No productivity losses**
				**• No societal perspective**
				• QALYs
Schechter et al. (2012) [40]	Payer	Program costs	**Program costs** (health educators labor costs, supervision labor costs, costs training health educators, telephone costs)	**NO**
				**• No direct medical costs**
				**• No productivity losses**
				**• No societal perspective**
				**• No QALYs**
Schechter et al. (2016) [39]	Payer	Program costs	**Program costs** (labor costs, telephone charges, incentives and printed materials, facilities and equipment)	**NO**
				• No direct medical costs
				**• No productivity losses**
				**• No societal perspective**
				**• No QALYs**
Gilmer et al. (2005) [29]	Payer	Direct costs of diabetes care	**Medical costs of diabetes care** (inpatient costs, outpatient costs, emergency visits and diabetes-related medications and supplies)	**NO**
				• Direct medical costs
				**• No productivity losses**
				**• No QALYs**
				**• No societal perspective**
Gilmer et al. (2007) [30]	Payer	Direct medical costs of diabetes care	**Medical costs of diabetes care** (costs of visits to RN and dieticians, participation in group classes, administrative overhead (visit scheduling, coordination of care with primary care provider, management of referrals, and support of database registry), cost of medicines and supplies, inpatient costs, outpatient costs, emergency room visit costs)	**NO**
				• Direct medical costs
				**• No productivity losses**
				• QALYs
				**• No societal perspective**
Huang et al. (2007) [33]	Societal	Program costs and direct medical costs of diabetes care	**Program costs**; **costs of diabetes care and its complications**	**NO**
				• Direct medical costs
				** • No productivity losses**
				• QALYs
				• Societal perspective
Banister et al. (2004) [37]	Payer	Program costs	**Program costs** (costs for dietician, costs for certified diabetes educator, costs for glucometer starter kit, cost for testing strips, rent/utilities, miscellaneous costs)	**NO**
				**• No direct medical costs**
				**• No productivity losses**
				**• No QALYs**
				**• No societal perspective**
Brownson et al. (2009) [34]	Payer	Setup and program costs, treatment costs, and complication costs	**Program and setup costs** (personnel costs, costs for contracted services, printing, supplies, other office costs, equipment, computing costs, overhead allocations); **treatment costs**; **complication costs**	**NO**
				• Direct medical costs
				** • No productivity losses**
				• QALYs
				** • No societal perspective**

ATSM, Automated Telephone Self-Management; CoDE, Community Diabetes Education; RN, Registered Nurse; QALY, quality-adjusted life years.

Five interventions are based on strong evidence (Table 3) [34, 35, 40–42]. Six analyses were cost-effective [29, 30, 32–34, 42] and eight were very cost-effective [31, 33, 35–40]. The very cost-effective interventions based on strong evidence involved: 1) CHWs visiting patients for T2DM in low-income individuals [41]; 2) one-to-one diabetes education/management program along with usual care in low-income, uninsured, ethnic minorities [35]; and 3) health educator phone calls to discuss self-management in low-income, urban populations [40]. Cost-effective interventions based on strong evidence involved: 1) interactive phone calls providing surveillance, education, and one-on-one counseling in low-income patients in safety-net clinics [42] (ongoing costs only); and 2) team of health educators in ethnic populations in disadvantaged areas with health disparities [34]. The interactive phone technology was only marginally cost-effective when both ongoing costs and start-up costs were considered [42]. There were no cost-ineffective interventions based on strong evidence.

Furthermore, five interventions are classified as very cost-effective, based on weak evidence: 1) monthly CHW visits in poor or medically indigent populations [31]; 2) diabetes self-management training in poor patients [37]; 3) Diabetes Prevention Program Lifestyle Intervention in Medicaid beneficiaries [36]; 4) Group Lifestyle Balance Program–group-based sessions for weight loss and raise physical activity in underserved populations [38]; and 5) telephone calls from health educators delivering behavioral counseling and self-management support in low-income populations [39]. Finally, three interventions based on weak evidence were valued as cost-effective: 1) CHW visits, health education, nutrition and exercise classes, and counseling sessions in low-income adults [32]; 2) nurse-led team with peer educators for low-income individuals [29, 30]; and 3) self-management support and group visits in patients of a community health center [33]. In studies supported by weak evidence, no interventions were classified as marginally cost-effective or cost-ineffective.

None of the 14 analyses reported all 24 points of the CHEERS guidelines, though most studies reported relatively well (Table 5). Six studies [30, 31, 33–36] failed to report on currency, price, date and conversion, while 5 studies [32, 34, 37, 41, 42] did not report on study parameters. Three studies [29, 31, 37] were not able to report on time horizon and discount rate. Most studies (86–93%) reported well on all other items. Four studies had a poor reporting quality, failing the purpose of CHEERS [33–35, 38]. Currently there is a lack of conformity to standard reporting checklists in this area of research (S1 Table). Furthermore, three of the eight cost-effectiveness studies with potential strong evidence were classified as weak, due to a less than excellent study quality (Table 3) [36, 38, 39]. Only one study [34] had external validity, reporting the sample was representative for the target population.

**Table 5:** Reporting on CHEERS guidelines.

CHEERS Item	Study does not report (n/14, %)	Study reports in compliance with CHEERS (n/14, %)	CHEERS item is N/A to study (n/14, %)
Title and abstract	1, 7.14%	13, 92.86%	-
Analytical model	1, 7.14%	13, 92.68%	-
Assumptions	1, 7.14%	12, 85.71%	1, 7.14%
Background and objectives	-	14, 100%	-
Characterizing heterogeneity	-	-	14, 100%
Characterizing uncertainty	3, 21.43%	11, 78.57%	-
Choice of health outcomes	2, 14.29%	12, 85.71	-
Choice of model	1, 7.14%	13, 92.86%	-
Comparators	1, 7.14%	13, 92.86%	-
Conflict of interest	1, 7.14%	13, 92.86%	-
Currency, price, date and conversion	6, 42.86%	4, 28.57%	4, 28.57%
Discount rate	1, 7.14%	10, 71.43%	3, 21.43%
Estimating resources and costs	2, 14.29%	12, 85.71%	-
Incremental costs and outcomes	-	14, 100%	-
Measurement and valuation of preference	-	1, 7.14%	13, 92.86%
Measurement of effectiveness	-	14, 100%	-
Setting and location	1, 7.14%	13, 92.86%	-
Source of funding	1, 7.14%	13, 92.86%	-
Study limitations	2, 14.29%	12, 85.715	-
Study parameters	5, 35.71%	9, 64.29%	-
Study perspective	3, 21.43%	11, 78.57%	-
Target populations and subgroup	2, 14.29%	12, 85.71%	-
Time horizon	3, 21.43%	11, 78.57%	-

### Characteristics of included patients and controls

Twelve papers excluded patients with T1DM or gestational diabetes, one intervention (2 studies) included both type 1 and 2 subjects [29, 30]. This intervention, Project Dulce, was considered in both articles, though the focus was on a different study design, making both studies relevant. These Project Dulce studies used a historical cohort enrolled prior to program implementation [29, 30]. Three studies [33, 36, 37] used no control group, but two of these studies performed a longitudinal pre-post analysis, assessing the cost-effectiveness before and after program implementation [33, 36]. One study assessed the cost-effectiveness through the use of a hypothetical control group [32]. Finally, the cost-effectiveness of eight studies was assessed by comparing an intervention with a control group [31, 34, 35, 38–42]. Of these, five studies [35, 39–42] were based on randomized controlled trial (RCT) data, while three studies [31, 34, 38] were based on other clinical data.

### Outcomes of the studies per category

#### Diabetes prevention in individuals at high risk

Two studies assessed the cost-effectiveness of T2DM prevention. Analyses demonstrate improvement with diabetes education/management [36, 38]. Roberts et al. [38], measured the effect as reduced metabolic syndrome risk at 1 year, while Gilmer et al. [36] used the average weight lost and improvement in HDL cholesterol. Both studies showed an increase in QALYs, though costs were also higher [36, 38]. The two studies calculate program costs and costs associated to diabetes care [36, 38], though one study [38] gives no information about which healthcare costs are included in the analysis. One study applied the societal perspective, but direct costs other than those of diabetes care and indirect costs are not included [38].

#### Diabetes management through CHWs

Four studies examined the economic impact of CHW interventions, compared to diabetes management without a CHW [31, 32, 35, 41]. A mean reduction in HbA1C in patients supported by CHWs was noted [31, 32, 35, 41]. Three studies demonstrated that CHW interventions reduced the risk of diabetes-related complications [31, 32, 35]. Costs were higher in the intervention group [31, 32, 35, 41], except for some sub-populations–patients aged 55–75 and males over a 20-year period showed cost-saving results [35]. Three studies included both the intervention and direct costs of care [32, 35, 41]. However, two studies give no information of which direct medical costs were considered in the analysis [32, 41]. One study gives an overview of the considered direct medical costs [41]. This study also considers the indirect costs, defined as time spent in the intervention or in using medical care [41]. Ryabov considers only program costs [31]. Two studies applied the payer perspective [31, 35], while two used the perspective of society [32, 41]. One of the latter studies failed to include direct and indirect costs other than those related to diabetes care [32].

#### Telephone-based diabetes management

Three studies investigated the cost-effectiveness of telephonic T2DM interventions [39, 40, 42], comparing it with usual T2DM self-management care without telephonic assistance. Handley et al. [42] noted an increase in quality-adjusted survival by using interactive phone calls providing surveillance, education, and one-on-one counseling, compared with usual care. Two studies found a significant decrease in HbA1C in the intervention group when comparing telephonic management plus print materials with print material only [39, 40]. Costs were higher in the intervention group [39, 40, 42]. All studies applied a payer perspective and assessed short-term results [39, 40, 42]. Also, the studies considered only program costs, not considering direct or indirect costs of diabetes care. The authors give an overview of costs included in the analysis [39, 40, 42].

#### Diabetes self-management training

Two studies assessed the cost-effectiveness of diabetes self-management training [34, 37], given by a team of health educators, nurses and dieticians; compared to usual diabetes self-management care. Brownson et al. showed an increase in life years and QALYs due to a reduced number and severity of diabetes-related complications [34]. Banister et al. demonstrated a mean decrease in HbA1C in the first 2–12 months after program entry [37]. The latter study considered only the intervention’s program costs [37], while a more complete picture of costs was given by Brownson et al., who assessed program and direct medical costs for treatment and complications [34]. The authors give no information about which medical costs are included in the analysis. Both studies applied the payer perspective, neglecting important direct and indirect costs related to diabetes care.

#### Nurse case management and peer education diabetes management

The cost-effectiveness of a nurse case and peer education T2DM intervention was examined by two Project Dulce studies [29, 30]. In the short-term, Gilmer et al., showed that 54% of participants achieved HbA1C values of less than 7%, compared to only 35% of controls [29]. Also, blood pressure and LDL cholesterol improved among the intervention group [29]. A second analysis, also conducted by Gilmer et al., assessed the intervention’s long-term outcomes for 4 cohorts: uninsured, governmental and commercial insurance [30]. Improvements in HbA1C and QALYs were found, being more favorable among the uninsured [30]. Costs were higher in the short and long-term [29, 30]. Both analyses applied the payer perspective, considering the direct medical costs. Both studies give an overview of cost categories included in the analysis [29, 30].

#### Quality improvement collaborative for diabetes

One study focused on the cost-effectiveness of a Quality Improvement Collaborative on glycemic control [33]. The program resulted in increased annual testing and the use and adherence to medications. A mean decrease in HbA1C, cholesterol, and improvements in QALYs were found. Lifetime complications were, therefore, expected to reduce on the longer term. The program increased total costs [33]. Program and direct medical costs were included, but an overview of the included cost categories was not reported. Although a societal perspective was applied, non-medical direct costs and indirect medical costs were absent.

## Discussion

This review suggests T2DM interventions are associated with improved health outcomes for underserved populations, with favorable cost-effectiveness. Almost all interventions were cost-effective or very cost-effective (ICER ≤ US$50,000); none of the studies were cost-saving. The evidence on which cost-effectiveness analyses were based varied. Nine out of 14 studies were based on strong evidence. Although the search covers a long period, from inception to December 21, 2020, the 14 studies that are included in the review are all relatively recent. The oldest paper has been published in 2004, while most included cost-effectiveness studies have been performed the last 10 years. This indicates the cost-effectiveness of diabetes management for underserved populations in the United States is a relatively new research area.

The World Health Organization endorses the use of cost-effectiveness analyses in advising policy-recommendations, and interventions with an ICER ≤ US$50,000/QALY are usually recommended [43]. Cost-effective interventions based on strong evidence should receive priority. However, for the studies included in this review, it remains extremely difficult to make recommendations about which interventions should be implemented for type 2 diabetes management in U.S. underserved populations. Policy recommendations are difficult to make since all interventions are cost-effective or very cost-effective, while only a handful are based on strong evidence and high quality. This highlights the urgent need for comprehensive and standardized cost-effectiveness studies. A standardized method for analysis is required to facilitate the task of assessing costs, ensuring comparability across studies as well as enhancing the generalizability of study findings. Indeed, significant attention has been given to the quality of the evidence [19], and the quality of reporting for the cost-effectiveness studies (CHEERS guidelines) [25]. The quality of the methodology in assessing the costs of interventions has been poorly assessed. Following the Recommendation of the Second Panel on Cost-Effectiveness in Health and Medicine [19], we note several limitations in the literature. Firstly, cost-effectiveness studies of T2DM in underserved populations are not transparent in showing which costs are included in their analyses. Secondly, every study applies its own methodology and costs for cost assessment. Non-standardized studies make cross-comparisons across interventions difficult. Therefore, the Second Panel on Cost-Effectiveness in Health and Medicine recommends quality-adjusted life years as an outcome measure, though their use is not always possible [19]. In those cases, condition-specific health outcomes should be used, which are easily interpretable for decision-makers [19]. However, surrogates endpoints should be avoided if possible (e.g. HbA1C), since they are not well linked to final endpoints [44]. Moreover, costs should be assessed from the societal perspective, meaning all direct and indirect costs should be included–regardless of who bears them [19]. This means not only the initial acquisition cost of a drug or intervention should be considered, but all other healthcare costs within the chosen time horizon should be included as well. If not, more expensive–but more effective–treatments risk being penalized and, consequently, wrongly rejected from consideration. Indirect productivity losses should also be included. However, to be useful, consistent and standardized methods need to be developed. Also, analyses need to be conducted from a long-term time horizon, including also future costs. In this review, four studies [33–35, 38] were unable to report a full description of which costs were considered, making it impossible to assess the quality of the cost-effectiveness result. A final shortcoming is related to the severity of diabetes, which can impact survival but–more importantly–quality of life. Therefore, it is important to assess all costs of T2DM [19], since an intervention–though costly–can have enormous impacts on the indirect costs, such as lost production, travel costs due to disease, complications and disabilities. Not considering these costs could lead to sub-optimal policy recommendations. It is, however, important to note that although more rigorous methodologies and more transparency is necessary, one size does not fit all. A first step to high-quality cost-effectiveness is, therefore, a comprehensive and standardized cost taxonomy–including all direct and indirect costs. Only then can the most optimal intervention for patients and society be guaranteed. Furthermore, the current focus on RCTs as a gold standard to deliver reliable evidence needs reconsideration. Indeed, RCTs often lack the necessary data on resource use and outcome assessment for cost-effectiveness. Next to the significant variation in methodology and reporting in cost-effectiveness analyses, the methods for the design, the conduct, and the analysis of data for economic analyses alongside clinical trials needs to be improved [45]. Also, the choice of the primary endpoint in a clinical study is unlikely an ideal endpoint for an economic evaluation [44]. To foster the use of comprehensive and standardized cost-effectiveness analyses, clinical trials should be designed to include not only clinical outcome measures, but also health resource use and health state utilities from the study patients [45]. Economic data should be fully collected as well to make trial-based cost-effectiveness studies possible [45].

Our systematic analysis has some limitations. First, we used the same threshold for all studies, regardless of whether ICERs were expressed as dollars per QALY or another outcome measure (such as dollars per percentage point decrease in HbA1C). QALYs consider patients’ quality of life, while other outcomes measures do not. The intervention’s impact on patients’ quality of life is hard to assess when studies do not express results as dollars per QALY, making it difficult to compare studies with different ICERs. Second, we excluded unpublished research, which may bias (publication bias) the results. Third, the review includes only a small number of studies. The two biggest drivers of limited research in this area is the urgent need for more resources to conduct cost-effectiveness studies as part of RCTs [45] as well as publication bias [46]. If the RCT is not adequately generalizable, also the cost-effectiveness study will suffer from this bias [46]. Therefore, only 14 studies met the inclusion criteria for this systematic review.

The strengths of this review include presenting a PRISMA reporting guideline-compliant review to guide current research in T2DM in underserved populations–clearly identifying limitations in current cost-effectiveness studies. Consequently, this review also serves as an important “policy agenda” to open the dialogue in economic research, to find the best way to improve future cost-effectiveness studies.

Further economic evaluations of T2DM interventions for vulnerable populations should consider the following. First, much greater emphasis needs to be placed on the scientific evidence for both the effectiveness and the cost-effectiveness of healthcare interventions to ensure patients receive the best possible care. The decision to use a particular intervention can be facilitated by identifying its cost-effectiveness in addition to its health benefits. Second, in cases where randomization is too difficult or impossible, a quasi-experimental non-randomized design is recommended [47]. Finally, future studies should focus on rigorously comparing the intervention group with a control group and a clear description of recruitment process should be given.

## Conclusions

The findings reported in this review are particularly timely given the high incidence of diabetes as well as the large and growing diabetes inequities in the United States. Prevalence of diabetes is particularly high among underserved individuals.

This review has shown the vast heterogeneity of cost calculation, and the urgent need for standardization. At present, there is no global consensus about the cost calculation in cost-effectiveness analyses. Therefore, studies use different techniques to account for the costs of the intervention. Cost calculation is currently non-standardized and causes difficulties when cross-comparing cost-effectiveness analyses. Future studies should make the necessary efforts to calculate costs from a societal perspective, independent from who bears the costs. This will provide robust and comprehensive cost-effectiveness analyses.

## Supporting information

S1 Checklist (DOC)

S1 File Search strategy. (DOCX)

S1 Table CHEERS checklist. (DOCX)

S2 Table Cost-effectiveness analyses for diabetes management interventions. (DOCX)

## References

[1] Centers for Disease Control and Prevention. National diabetes statistics report, 2017. Atlanta (GA): National Center for Chronic Disease Prevention and Health Promotion, Division of Diabetes Translation; 2017. Available from: https://dev.diabetes.org/sites/default/files/2019-06/cdc-statistics-report-2017.pdf

[2] American Diabetes Association. Economic Costs of Diabetes in the US in 2017. Diabetes Care. 2018;41: 917. doi: 10.2337/dci18-0007 29567642 PMC5911784

[3] Berkowitz SA, Meigs JB, DeWalt D, Seligman HK, Barnard LS, Bright O-JM, et al. Material need insecurities, control of diabetes mellitus, and use of health care resources: results of the Measuring Economic Insecurity in Diabetes study. JAMA Intern Med. 2015;175(2): 257–265. doi: 10.1001/jamainternmed.2014.6888 25545780 PMC4484589

[4] Crandall JP, Knowler WC, Kahn SE, Marrero D, Florez JC, Bray GA, et al. The prevention of type 2 diabetes. Nat Clin Pract Endocrinol Metab. 2008;4(7): 382–393. doi: 10.1038/ncpendmet0843 18493227 PMC2573045

[5] Diabetes Control and Complications Trial Research Group, Nathan DM, Genuth S, Lachin J, Cleary P, Crofford O, et al. The effect of intensive treatment of diabetes on the development and progression of long-term complications in insulin-dependent diabetes mellitus. N Engl J Med. 1993;329(14): 977–986. doi: 10.1056/NEJM199309303291401 8366922

[6] Remuzzi G, Schieppati A, Ruggenenti P. Nephropathy in patients with type 2 diabetes. N Engl J Med. 2002;346(15): 1145–1151. doi: 10.1056/NEJMcp011773 11948275

[7] Ref ESRD:United States Renal System. Healthcare expenditures for persons with ESRD—Google Search. Accessed June 29, 2020. https://adr.usrds.org/2020/end-stage-renal-disease/9-healthcare-expenditures-for-persons-with-esrd

[8] Turner RC, Cull CA, Frighi V, Holman RR, UK Prospective Diabetes Study (UKPDS) Group. Glycemic control with diet, sulfonylurea, metformin, or insulin in patients with type 2 diabetes mellitus: progressive requirement for multiple therapies (UKPDS 49). JAMA. 1999;281(21): 2005–2012. doi: 10.1001/jama.281.21.2005 10359389

[9] https://www.kumc.edu/school-of-medicine/psychiatry-and-behavioral-sciences/clinical-psychology-training-programs/predoctoral-internship-program/underserved-populations.html

[10] Li R, Zhang P, Barker LE, Chowdhury FM, Zhang X. Cost-effectiveness of interventions to prevent and control diabetes mellitus: a systematic review. Diabetes Care. 2010;33(8):1872–1894. doi: 10.2337/dc10-0843 20668156 PMC2909081

[11] Zhong Y, Lin P-J, Cohen JT, Winn AN, Neumann PJ. Cost-utility analyses in diabetes: a systematic review and implications from real-world evidence. Value Health. 2015;18(2): 308–314. doi: 10.1016/j.jval.2014.12.004 25773567

[12] Hong D, Si L, Jiang M, Shao H, Ming W-K, Zhao Y, et al. Cost effectiveness of sodium-glucose cotransporter-2 (SGLT2) inhibitors, glucagon-like peptide-1 (GLP-1) receptor agonists, and dipeptidyl peptidase-4 (DPP-4) inhibitors: a systematic review. Pharmacoeconomics. 2019;37(6): 777–818. doi: 10.1007/s40273-019-00774-9 30854589

[13] Zhou X, Siegel KR, Ng BP, Jawanda S, Proia KK, Zhang X, et al. Cost-effectiveness of diabetes prevention interventions targeting high-risk individuals and whole populations: a systematic review. Diabetes Care. 2020;43(7): 1593–1616. doi: 10.2337/dci20-0018 33534726

[14] Siegel KR, Ali MK, Zhou X, Ng BP, Jawanda S, Proia K, et al. Cost-effectiveness of interventions to manage diabetes: has the evidence changed since 2008? Diabetes Care. 2020;43(7): 1557–1592. doi: 10.2337/dci20-0017 33534729

[15] Liberati A, Altman DG, Tetzlaff J, Mulrow C, Gøtzsche PC, Ioannidis JPA, et al. The PRISMA statement for reporting systematic reviews and meta-analyses of studies that evaluate health care interventions: explanation and elaboration. J Clin Epidemiol. 2009;62(10): e1–e34. doi: 10.1016/j.jclinepi.2009.06.006 19631507

[16] Yon Y, Mikton CR, Gassoumis ZD, Wilber KH. Elder abuse prevalence in community settings: a systematic review and meta-analysis. Lancet Glob Health. 2017;5(2): e147–e156. doi: 10.1016/S2214-109X(17)30006-2 28104184

[17] Berlin JA, Miles CG, Cirigliano MD, Conill AM, Goldmann DR, Horowitz DA, et al. Does blinding of readers affect the results of meta-analyses? Results of a randomized trial. Online J Curr Clin Trials. 1997;6(1). Available from: https://access.portico.org/stable?au=phzcxbjgq

[18] Irwig L, Tosteson AN, Gatsonis C, Lau J, Colditz G, Chalmers TC, et al. Guidelines for meta-analyses evaluating diagnostic tests. Ann Intern Med. 1994;120(8): 667–676. doi: 10.7326/0003-4819-120-8-199404150-00008 8135452

[19] Carias C, Chesson HW, Grosse SD, Li R, Meltzer MI, Miller GF, et al. Recommendations of the second panel on cost effectiveness in health and medicine: a reference, not a rule book. Am J Prev Med. 2018;54(4): 600–602. doi: 10.1016/j.amepre.2017.11.013 29338958 PMC6038124

[20] U.S. Bureau of Labor Statistics. Consumer Price Index Archived News Releases—December 2019. Available from: https://www.bls.gov/news.release/archives/cpi_01142020.htm

[21] Grosse SD. Assessing cost-effectiveness in healthcare: history of the $50,000 per QALY threshold. Expert Rev Pharmacoecon Outcomes Res. 2008;8(2): 165–178. doi: 10.1586/14737167.8.2.165 20528406

[22] Djulbegovic B, Trikalinos TA, Roback J, Chen R, Guyatt G. Impact of quality of evidence on the strength of recommendations: an empirical study. BMC Health Serv Res. 2009;9(1): 120. doi: 10.1186/1472-6963-9-120 19622148 PMC2722589

[23] Atkins D, Best D, Briss PA, Eccles M, Falck-Ytter Y, Flottorp S, et al. Grading quality of evidence and strength of recommendations. BMJ. 2004;328(7454): 1490. doi: 10.1136/bmj.328.7454.1490 15205295 PMC428525

[24] American Diabetes Association. Introduction: Standards of Medical Care in Diabetes—2020. Diabetes Care. 2020;43(suppl 1): S1–S2. doi: 10.2337/dc20-Sint 31862741

[25] Husereau D, Drummond M, Petrou S, Carswell C, Moher D, Greenberg D, et al. Consolidated health economic evaluation reporting standards (CHEERS) statement. Cost Eff Resour Alloc. 2013;11(1): 6. doi: 10.1186/1478-7547-11-6 23531194 PMC3607888

[26] Alderson P, Green S, Higgins JPT, editors. Cochrane Reviewers’ Handbook 4.2.2 [updated March 2004]. Chichester, UK: John Wiley & Sons; 2004. Available from: https://www.iecs.org.ar/cochrane/guias/Handbook_4-2-2.pdf

[27] Norris SL, Engelgau MM, Narayan KV. Effectiveness of self-management training in type 2 diabetes: a systematic review of randomized controlled trials. Diabetes Care. 2001;24(3): 561–587. doi: 10.2337/diacare.24.3.561 11289485

[28] Klonoff DC, Schwartz DM. An economic analysis of interventions for diabetes. Diabetes Care. 2000;23(3): 390–404. doi: 10.2337/diacare.23.3.390 10868871

[29] Gilmer TP, Philis-Tsimikas A, Walker C. Outcomes of Project Dulce: a culturally specific diabetes management program. Ann Pharmacother. 2005;39(5): 817–822. doi: 10.1345/aph.1E583 15769828

[30] Gilmer TP, Roze S, Valentine WJ, Emy-Albrecht K, Ray JA, Cobden D, et al. Cost-effectiveness of diabetes case management for low-income populations. Health Serv Res. 2007;42(5): 1943–1959. doi: 10.1111/j.1475-6773.2007.00701.x 17850527 PMC2254564

[31] Ryabov I. Cost-effectiveness of Community Health Workers in controlling diabetes epidemic on the US–Mexico border. Public Health. 2014;128(7): 636–642. doi: 10.1016/j.puhe.2014.05.002 24999158

[32] Brown HS III, Wilson KJ, Pagán JA, Arcari CM, Martinez M, Smith K, et al. Cost-effectiveness analysis of a community health worker intervention for low-income Hispanic adults with diabetes. Prev Chronic Dis. 2012;9: E140. doi: 10.5888/pcd9.120074 22916995 PMC3475531

[33] Huang ES, Zhang Q, Brown SE, Drum ML, Meltzer DO, Chin MH. The cost-effectiveness of improving diabetes care in US federally qualified community health centers. Health Serv Res. 2007;42(6 Pt 1): 2174–2193. doi: 10.1111/j.1475-6773.2007.00734.x 17995559 PMC2151395

[34] Brownson CA, Hoerger TJ, Fisher EB, Kilpatrick KE. Cost-effectiveness of diabetes self-management programs in community primary care settings. Diabetes Educ. 2009;35(5): 761–769. doi: 10.1177/0145721709340931 19622716

[35] Prezio EA, Pagán JA, Shuval K, Culica D. The Community Diabetes Education (CoDE) program: cost-effectiveness and health outcomes. Am J Prev Med. 2014;47(6): 771–779. doi: 10.1016/j.amepre.2014.08.016 25455119

[36] Gilmer T, O’Connor PJ, Schiff JS, Taylor G, Vazquez-Benitez G, Garrett JE, et al. Cost-effectiveness of a community-based diabetes prevention program with participation incentives for Medicaid beneficiaries. Health Serv Res. 2018;53(6): 4704–4724. doi: 10.1111/1475-6773.12973 29770445 PMC6232439

[37] Banister NA, Jastrow ST, Hodges V, Loop R, Gillham MB. Diabetes self-management training program in a community clinic improves patient outcomes at modest cost. J Am Diet Assoc. 2004;104(5): 807–810. doi: 10.1016/j.jada.2004.02.027 15127069

[38] Roberts MS, Kramer MK, Orchard TJ, Piatt GA. Cost-effectiveness analysis of efforts to reduce risk of type 2 diabetes and cardiovascular disease in southwestern Pennsylvania, 2005–2007. Prev Chronic Dis. 2010;7(5): A109. 20712936 PMC2938403

[39] Schechter CB, Walker EA, Ortega FM, Chamany S, Silver LD. Costs and effects of a telephonic diabetes self-management support intervention using health educators. J Diabetes Complications. 2016;30(2): 300–305. doi: 10.1016/j.jdiacomp.2015.11.017 26750743 PMC4761277

[40] Schechter CB, Cohen HW, Shmukler C, Walker EA. Intervention costs and cost-effectiveness of a successful telephonic intervention to promote diabetes control. Diabetes Care. 2012;35(11): 2156–2160. doi: 10.2337/dc12-0048 22851599 PMC3476890

[41] Huang SJ, Galárraga O, Smith KA, Fuimaono S, McGarvey ST. Cost-effectiveness analysis of a cluster-randomized, culturally tailored, community health worker home-visiting diabetes intervention versus standard care in American Samoa. Hum Resour Health. 2019;17(1): 17. doi: 10.1186/s12960-019-0356-6 30836964 PMC6402127

[42] Handley MA, Shumway M, Schillinger D. Cost-effectiveness of automated telephone self-management support with nurse care management among patients with diabetes. Ann Fam Med. 2008;6(6): 512–518. doi: 10.1370/afm.889 19001303 PMC2582479

[43] Laupacis A, Feeny D, Detsky AS, Tugwell PX. How attractive does a new technology have to be to warrant adoption and utilization? Tentative guidelines for using clinical and economic evaluations. CMAJ. 1992;146(4): 473–481. 1306034 PMC1488412

[44] Knowler WC, Barrett-Connor E, Fowler SE, Hamman RF, Lachin JM, Walker EA, et al. Reduction in the incidence of type 2 diabetes with lifestyle intervention or metformin. N Engl J Med. 2002;346(6): 393–403. doi: 10.1056/NEJMoa012512 11832527 PMC1370926

[45] Ramsey SD, Willke RJ, Glick H, Reed SD, Augustovski F, Jonsson B, et al. Cost-effectiveness analysis alongside clinical trials II—An ISPOR Good Research Practice Task Force Report. Value Health. 2015;18: 161–172. doi: 10.1016/j.jval.2015.02.001 25773551

[46] Weintraub WS, Cohen DJ. The limits of cost-effectiveness analysis. Circ Cardiovasc Qual Outcomes. 2009;2: 55–58. doi: 10.1161/CIRCOUTCOMES.108.812321 20031813

[47] Schianca GPC, Rossi A, Sainaghi PP, Maduli E, Bartoli E. The significance of impaired fasting glucose versus impaired glucose tolerance: importance of insulin secretion and resistance. Diabetes Care. 2003;26(5): 1333–1337. doi: 10.2337/diacare.26.5.1333 12716784

