# Supplementary material for: Comprehensive cost-effectiveness of diabetes management for the underserved in the United States: A systematic review
Source: PLoS One. 2021 Nov 18;16(11):e0260139. doi: 10.1371/journal.pone.0260139 (PMC8601459; doi:10.1371/journal.pone.0260139)
Supplement: S1 File — (DOCX) [file pone.0260139.s002.docx]

S1 File. Search Strategy.

Database: Ovid MEDLINE ® ALL <1946 to January 20, 2020

1. Exp DIABETES MELLITUS/ (392203)
2. Diabet*.sh,ti,ab. (622150)
3. Or/1-2 (624127)
4. Exp Preventive medicine/ (34715)
5. Exp Therapeutics/ (4243634)
6. (therap* or treat* or prevent*).fs,ti,ab. (8743215
7. Or/4-6 (10425469)
8. Exp MEDICALLY UNDERSERVED AREA/ or exp Health Services Accessibility/ (107166)
9. Exp Medically Uninsured/ (7026)
10. (underserv* or uninsur* or underinsur*).ti,ab. (15860)
11. Or/8-10 (120748)
12. Exp “Costs and Cost Analysis”/ (219972)
13. Exp “Cost of illness”/ (24249)
14. Exp “Cost Savings”/ (10974)
15. Exp “Cost Allocation”/ (1989)
16. Exp “Cost Control”/ (32042)
17. Exp Cost-Benefit Analysis/ (74686)
18. Exp Health Care Costs/ (59735)
19. Exp Direct Service Costs/ (1151)
20. Exp Hospital Costs/ (10057)
21. Exp Health Expenditures/ (20018)
22. Exp Employer Health Costs/ (1088)
23. Exp Drug Costs/ (14956)
24. ((cost* or budget* or expenditure* or economic*) adj1 (benefit* or analysis* or expenditure* or effective*)).ti,ab. (188141)
25. Exp Economic, Pharmaceutical/ or exp Economics, Medical/ or exp Economics, Hospital/ or exp Economics, Nursing/ (43361)
26. Or/12-25 (371571)
27. 3 and 7 and 11 and 28 (264)
