# Supplementary material for: Comprehensive cost-effectiveness of diabetes management for the underserved in the United States: A systematic review
Source: PLoS One. 2021 Nov 18;16(11):e0260139. doi: 10.1371/journal.pone.0260139 (PMC8601459; doi:10.1371/journal.pone.0260139)
Supplement: S1 Table — (DOCX) [file pone.0260139.s003.docx]

**S1 Table. CHEERS Checklist***

| **Item** | **Gilmer (2018)^35**^** | **Roberts (2010)^37^** | **Brown (2012)^31^** | **Huang (2019)^40^** | **Prezio (2014)^34^** | **Ryabov (2014)^30^** | **Handley (2008)^41^** | **Schechter (2012)^39^** | **Schechter (2016)^38^** | **Gilmer (2005)^28^** | **Gilmer (2007)^29^** | **Huang (2007)^32^** | **Banister (2004)^36^** | **Brownson (2009)^33^** |
| --- | --- | --- | --- | --- | --- | --- | --- | --- | --- | --- | --- | --- | --- | --- |
| Title | **◆** | **◆** | **◆** | **◆** | **◆** | **◆** | **◆** | **◆** | **◆** | **X** | **◆** | **◆** | **◆** | **◆** |
| Abstract | **◆** | **◆** | **◆** | **◆** | **◆** | **◆** | **◆** | **◆** | **◆** | **◆** | **◆** | **◆** | **X** | **◆** |
| Background and objectives | **◆** | **◆** | **◆** | **◆** | **◆** | **◆** | **◆** | **◆** | **◆** | **◆** | **◆** | **◆** | **◆** | **◆** |
| Target population and subgroups | **◆** | **◆** | **◆** | **◆** | **◆** | **◆** | **◆** | **◆** | **X** | **◆** | **◆** | **X** | **◆** | **◆** |
| Setting and location | **◆** | **◆** | **◆** | **◆** | **◆** | **◆** | **◆** | **◆** | **X** | ◆ | **◆** | **◆** | **◆** | **◆** |
| Study perspective | **◆** | **◆** | **◆** | **◆** | **◆** | **X** | **◆** | **◆** | **◆** | **◆** | **◆** | **◆** | **X** | **X** |
| Comparators | **◆** | **◆** | **◆** | **◆** | **◆** | **◆** | **◆** | **◆** | **◆** | **◆** | **◆** | **◆** | **X** | **◆** |
| Time horizon | **◆** | **◆** | **◆** | **◆** | **◆** | **X** | **◆** | **◆** | **◆** | **X** | **◆** | **◆** | **X** | **◆** |
| Discount rate | **◆** | **◆** | **◆** | **◆** | **◆** | **X** | **N/A** | **◆** | **◆** | **N/A** | **◆** | **◆** | **N/A** | **◆** |
| Choice of health outcomes | **◆** | **X** | **◆** | **◆** | **◆** | **◆** | **◆** | **◆** | **◆** | **◆** | **X** | **◆** | **◆** | **◆** |
| Measurement of effectiveness | **◆** | **◆** | **◆** | **◆** | **◆** | **◆** | **◆** | **◆** | **◆** | **◆** | **◆** | **◆** | **◆** | **◆** |
| Measurement and valuation of preference based outcomes | **◆** | **N/A** | **N/A** | **N/A** | **N/A** | **N/A** | **N/A** | **N/A** | **N/A** | **N/A** | **N/A** | **N/A** | **N/A** | **N/A** |
| Estimating resources and costs | **◆** | **X** | **◆** | **◆** | **◆** | **X** | **◆** | **◆** | **◆** | **◆** | **◆** | **◆** | **◆** | **◆** |
| Currency, price date, and conversion | **X** | **◆** | **◆** | **◆** | **X** | **X** | **N/A** | **N/A** | **N/A** | **◆** | **X** | **X** | **N/A** | **X** |
| Choice of model | **◆** | **◆** | **◆** | **◆** | **◆** | **◆** | **◆** | **◆** | **◆** | **◆** | **◆** | **◆** | **X** | **◆** |
| Assumptions | **X** | **◆** | **◆** | **◆** | **◆** | **◆** | **◆** | **◆** | **◆** | **◆** | **◆** | **◆** | **N/A** | **◆** |
| Analytical model | **X** | **X** | **X** | **X** | **X** | **X** | **X** | **X** | **X** | **◆** | **X** | **X** | **X** | **X** |
| Study parameters | **◆** | **◆** | **X** | **X** | **◆** | **◆** | **X** | **◆** | **◆** | **◆** | **◆** | ◆ | **X** | **X** |
| Incremental costs and outcomes | **◆** | **◆** | **◆** | **◆** | **◆** | **◆** | **◆** | **◆** | **◆** | **◆** | **◆** | **◆** | **◆** | **◆** |
| Characterizing uncertainty | **◆** | **◆** | **◆** | **◆** | **◆** | **X** | **◆** | **◆** | **◆** | **X** | **◆** | **◆** | **X** | **◆** |
| Characterizing heterogeneity | **N/A** | **N/A** | **N/A** | **N/A** | **N/A** | **N/A** | **N/A** | **N/A** | **N/A** | **N/A** | **N/A** | **N/A** | **N/A** | **N/A** |
| Study findings, limitations, generalizability and current knowledge | **◆** | **◆** | **◆** | **◆** | **◆** | **◆** | **◆** | **X** | **◆** | **◆** | **◆** | **◆** | **X** | **◆** |
| Source of funding | **◆** | **◆** | **◆** | **◆** | **◆** | **◆** | **◆** | **◆** | **◆** | **X** | **◆** | **◆** | **◆** | **◆** |
| Conflict of interest | **◆** | **◆** | **◆** | **◆** | **◆** | **◆** | **◆** | **◆** | **◆** | **X** | **◆** | **◆** | **◆** | **◆** |
| *For description of items, see: Husereau D, Drummond M, Petrou S, Greenberg D, Augustovski F, Briggs AH, et al. Consolidated Health Economic Evaluation Reporting Standards (CHEERS) statement. BMJ. 2013;346: f1049. doi: 10.1136/bmj.f1049.  **See main paper for citations to studies included in the systematic review.  Legend: **◆** = CHEERS reporting item satisfied by study; **X** = reporting item not satisfied; **N/A** = reporting item is not applicable to the study. | | | | | | | | | | | | | | |
