# Supplementary material for: Comprehensive cost-effectiveness of diabetes management for the underserved in the United States: A systematic review
Source: PLoS One. 2021 Nov 18;16(11):e0260139. doi: 10.1371/journal.pone.0260139 (PMC8601459; doi:10.1371/journal.pone.0260139)
Supplement: S2 Table — (DOCX) [file pone.0260139.s004.docx]

**S2 Table. Cost-effectiveness Analyses for Diabetes Management Interventions**

| **Source and state and (reference)** | **Study population** | **Intervention** | **Control** | **Effectiveness data and outcomes** | **Analytical horizon and discount rate** | **Costs included** | **Economic outcomes** | **Perspective** | **Cost-effectiveness results** |
| --- | --- | --- | --- | --- | --- | --- | --- | --- | --- |
| *Diabetes prevention in high-risk populations* | | | | | | | | | |
| Gilmer et al. (2018)^35^ | Medicaid beneficiaries at high risk for type 2 diabetes | Diabetes Prevention Program Lifestyle Intervention adapted for community settings - Intensive group-based lifestyle intervention | No control | Change in weight, cholesterol, HbA1C, BP, and QALYs | 40-year time horizon (3%) though a validated diabetes simulation model. | Program costs (program coaching costs [staffing costs and costs for delivering the program curriculum], clinic costs [cost of program materials - measuring cups, exercise band, home scales, paper and other materials, educational materials, resources and services to support participation such as transportation and childcare], financial incentive costs [participation and goal-based incentive provided to program participants]), health care costs (costs for profession, outpatient, and pharmacy services, costs of complications) | Intervention costs, healthcare costs | Payer | US$14,011/QALY |
| Roberts at al. (2010)^37^ | Urban, medically underserved population | Group Lifestyle Balance Program– Group-based sessions to achieve and maintain weight loss and to progressively raise activity levels to 150 minutes per week of moderately intense physical activity | Usual care – absence of screening program | Changes in BMI, waist circumference, HDL, fasting glucose, BP, and QALYs | 3-year time horizon (3%) though a Markov decision model | Program costs (costs of screening, personnel costs),no info about costs of diabetes | Costs of diabetes and program costs | Societal | US$3,420/QALY |
| *Diabetes management in patients diagnosed with type 2 diabetes* | | | | | | | | | |
| Diabetes management through CHWs | | | | | | | | | |
| Brown et al. (2012)^31^ | Low-income Hispanic adults | Home-based CHW visits, classroom health education classes, nutrition classes, exercise classes, and counseling sessions. | Hypothetical usual care | Change in HbA1C, complications and QALYs | 20-year time horizon (3%) through the Archimedes model | Program costs (staff and volunteer time, participant time, materials, transport costs, fixed cost per class, CHW training cost), direct medical costs, and lifestyle change costs | All measurable opportunity costs | Societal | ICER: US$33,319/QALY  ICER for ages 50-65:  US$30,786/QALY  ICER when HbA1C>9%  and lowered below  9% at follow-up:  US$10,995/QALY |
| Huang et al. (2019)^40^ | Samoan population, low-income | Community health workers visiting patients at home for diabetes management | Waitlisted and standard care | Changes in HbA1C, medication adherence, healthy eating, physical activity | 2 year time horizon (N/A) | Intervention costs (start-up capital costs, staff salaries, donated space, other overhead costs), costs for diabetes care (clinic and hospital ambulatory costs, hospital ED costs, hospital inpatient costs, hospital procedure costs, patient indirect costs) | Intervention costs and costs of diabetes care | Societal | ICER per reduction in  HbA1C point:  US$1,121.26/reduction  of 1 point in HbA1C tho  ICER per QALY gained:  US$13,191 |
| Prezio et al. (2014)^34^ | Low-income, uninsured, ethnic minority populations | Received a one-to-one culturally tailored diabetes education and management program along with usual care | Usual care | Change in HbA1C, BP, height, weight, and QALYs | 20 year time horizon (3%) through the Archimedes model | Staff and participant time, supplies for CoDE program, medical costs | Program costs and medical costs | Payer | ICER over 20 years:  US$355/QALY  ICER age 55-75:  Cost-saving  ICER women:  US$1,320/QALY  ICER men:  Cost-saving |
| Ryabov (2014)^30^ | Poor or medically indigent immigrant population | Received monthly visits from community health workers | Usual care | Change in HbA1C, total cholesterol, HDL, triglycerides, diastolic BP, systolic BP, and BMI | Lifetime time horizon (3%) though the CDC Diabetes Cost-Effectiveness Model | Cost of implementing intervention (wages and office operations), records of materials and equipment used | Program costs | Payer | ICER: US$13,810/QALY |
| Diabetes management through telephonic intervention | | | | | | | | | |
| Handley et al. (2008)^41^ | Low-income patients in safety-net clinics | Interactive phone technology to provide surveillance, patient education, and one-on-one counseling | Usual care | QALYs | 1 year time horizon | Program costs (nurse care manager training, development of ATSM messages, translation and recording of messages in 3 languages, programming setup costs, patient recruitment and follow-up time, fixed monthly ATSM maintenance, costs associated with outgoing weekly ATSM calls, and direct nurse telephone care management with patients, and overhead costs) | Program costs | Payer | ICER start-up +  ongoing:  US$65,167/QALY  ICER ongoing:  US$32,333/QALY |
| Schechter et al. (2012)^39^ | Low-income, urban populations | Health educator for up to 10 self-management support phone calls to discuss self-management as found in the print materials mailed to them | Only print materials | Change in HbA1C, the mean decrease in HbA1C between the beginning and end of the intervention, the proportion of participants achieving a target HbA1C value of <7% | 1 year time horizon | Program costs (health educators labor costs, supervision labor costs, costs training health educators, telephone costs) | Program costs | Payer | ICER per percentage  point decrease in HbA1C:  US$490.58/percentage  point decrease in  HbA1C  ICER per additional  person achieving  HbA1C goal:  US$2,617.35/additional  person achieving  HbA1C goal |
| Schechter et al. (2016)^38^ | Low-income population | 4 or 8 telephone calls over 12 months, depending on HbA1C level, from trained, supervised health educators to deliver behavioral counseling and self-management support, in addition to the print material | Print material only | Aggregate change in HbA1C, number of people decreasing HbA1C by 0.5 points and 1 point | 1 year time horizon | Program costs (labor costs, telephone charges, incentives and printed materials, facilities and equipment) | Program costs | Payer | ICER for expanding an  existing program:  US$464.41/percentage  point HbA1C  US$2,109.25/1%  decrease in HbA1C  US$1,767.42/0.5%  decrease in HbA1C |
| Nurse case management and peer education diabetes management | | | | | | | | | |
| Gilmer et al. (2005)^28^ | Low-income, underinsured Latinx | Nurse-led team with registered nurse, certified diabetes educator, medical assistant and dietician. The goal is to meet the ADA standards of care and achieve improvements in HbA1C, BP and lipid parameters. In addition, the program offers group self-management training (8 weeks) led by trained peer educators | Historical cohort of patients enrolled prior to the implementation of Project Dulce | Changes in HbA1C, blood pressure and lipid parameters | 1 year time horizon | Direct costs (inpatients, outpatient, emergency visits and diabetes-related medications and supplies) | Direct costs | Payer | Incremental cost:  US$1,346, while 54% of  patients had HbA1C<7%  (compared to 35%); 68%  of patients had BP<  130/80mmHG  (compared to 49%);  54% of patients had  LDL values<100mg/dL  (compared to 18%) |
| Gilmer et al. (2007)^29^ | Low-income, underinsured Latinx | Nurse-led team with registered nurse, certified diabetes educator, medical assistant and dietician. The goal is to meet the ADA standards of care and achieve improvements in HbA1C, BP and lipid parameters. In addition, the program offers group self-management training (8 weeks) led by trained peer educators | Historical cohort of patients enrolled prior to the implementation of Project Dulce | Life expectancy, quality-adjusted life expectancy, cumulative incidence of complications | 40 year time horizon (3%) through the Center for Outcomes Research Diabetes Model | Direct medical costs (costs of visits to RN and dieticians, participation in group classes, administrative overhead [visit scheduling, coordination of care with PCP, management of referrals, and support of database registry], cost of medicines and supplies, inpatient and outpatient costs, emergency room visit costs) | Direct medical costs | Payer | ICER uninsured:  US$10,141/QALY  ICER County Medical  Services:  US$24,584/QALY  ICER Medi-Cal:  US$44,941/QALY  ICER commercial:  US$69,587/QALY |
| Quality Improvement Collaborative Diabetes | | | | | | | | | |
| Huang et al. (2007)^32^ | Patients of a community health center | Patients received self-management support and group visits | No control | Change in HbA1C, total cholesterol, BP, diabetes complications, QALYs | Lifetime time horizon (3%) through a Markov Monte Carlo simulation model | Program costs, costs of diabetes care and its complications | Direct medical costs and program costs | Societal | ICER:US$33,386/QALY  ICER ACE inhibitors:  US$23,653/QALY  ICER glucose control and  associated testing:  US$104,811/QALY  ICER cholesterol testing:  US$416,850/QALY  ICER aspirin:  US$151,767/QALY |
| Diabetes self-management training | | | | | | | | | |
| Banister et al. (2004)^36^ | Patients below the US Federal poverty level | Diabetes self-management training program with group classes and individual dietician consults | No control | Change in body weight, prescribed diabetes medications, HbA1C, attendance | 1 year time horizon | Costs for dietician, costs for certified diabetes educator, costs for glucometer starter kit, cost for testing strips, rent/utilities, miscellaneous costs | Program costs | Payer | ICER: US$185/  average decrease of  HbA1C of 1.5 points |
| Brownson et al. (2009)^33^ | Variety of ethnic populations in disadvantaged areas with notable health disparities | Various approaches are used in the different communities to reach and engage their respective patient populations in self-management | Usual care | Change in HbA1C, BP, lipids , complications, and QALYs | Lifetime time horizon (3%) through the CDC Diabetes Cost-Effectiveness Model | Operating costs (personnel costs, costs for contracted services, printing, supplies, other office costs, equipment, computing costs, overhead allocations) | Setup and program costs, treatment costs, and complication costs | Payer | ICER: US$39,563/QALY |
